# Biotechnological Potential of LSD1, EDS1, and PAD4 in the Improvement of Crops and Industrial Plants

**DOI:** 10.3390/plants8080290

**Published:** 2019-08-16

**Authors:** Maciej Jerzy Bernacki, Weronika Czarnocka, Magdalena Szechyńska-Hebda, Ron Mittler, Stanisław Karpiński

**Affiliations:** 1 Department of Plant Genetics, Breeding and Biotechnology, Faculty of Horticulture, Biotechnology and Landscape Architecture, Warsaw University of Life Sciences, Nowoursynowska Street 159, 02-776 Warsaw, Poland; 2 The Division of Plant Sciences, College of Agriculture, Food and Natural Resources, Christopher S. Bond Life Sciences Center, University of Missouri, Columbia, MO 65201, USA; 3 Department of Botany, Faculty of Agriculture and Biology, Warsaw University of Life Sciences, Nowoursynowska Street 159, 02-776 Warsaw, Poland; 4 The Franciszek Górski Institute of Plant Physiology, Polish Academy of Sciences, Niezapominajek Street 21, 30-239 Cracow, Poland; 5 The Plant Breeding and Acclimatization Institute - National Research Institute, 05-870 Błonie, Radzików, Poland

**Keywords:** Biomass, LSD1, EDS1, PAD4, seed yield, WUE

## Abstract

Lesion Simulating Disease 1 (LSD1), Enhanced Disease Susceptibility (EDS1) and Phytoalexin Deficient 4 (PAD4) were discovered a quarter century ago as regulators of programmed cell death and biotic stress responses in *Arabidopsis thaliana*. Recent studies have demonstrated that these proteins are also required for acclimation responses to various abiotic stresses, such as high light, UV radiation, drought and cold, and that their function is mediated through secondary messengers, such as salicylic acid (SA), reactive oxygen species (ROS), ethylene (ET) and other signaling molecules. Furthermore, LSD1, EDS1 and PAD4 were recently shown to be involved in the modification of cell walls, and the regulation of seed yield, biomass production and water use efficiency. The function of these proteins was not only demonstrated in model plants, such as *Arabidopsis thaliana* or *Nicotiana benthamiana,* but also in the woody plant *Populus tremula x tremuloides*. In addition, orthologs of LSD1, EDS1, and PAD4 were found in other plant species, including different crop species. In this review, we focus on specific LSD1, EDS1 and PAD4 features that make them potentially important for agricultural and industrial use.

## 1. Introduction

Enhancing agricultural production is one of the greatest challenges of the 21st century. This goal should be achieved with the least possible use of pesticides and fertilizers, that despite their benefits, can contaminate groundwater and the surrounding soils, and are highly hazardous to both humans and the environment [1,2,3,4,5]. Moreover, along with the need for increasing yield quantity and quality, a major challenge for modern agriculture is the reduction of water consumption, as a high proportion of available freshwater from rivers and groundwater goes into crop irrigation, and the availability of these waters is rapidly decreasing, due to global climatic changes and dwindling water resources [6,7,8,9].

For three decades, genetic engineering has made it possible to develop genetically-modified (GM) crops and trees designed for yield improvement and efficient agriculture management [10,11,12,13]. GM crops were introduced into commercial use in the USA in 1994 [14]. To date, soybean, cotton and corn are the most common genetically-modified crops [15]. Nearly 93% of soybean and 88% of corn crops in the US are genetically modified, according to the United States Food and Drug Administration (FDA). Many GM crops have been engineered to resist certain herbicides, e.g., the majority of currently-cultivated GM plants are resistant to the herbicide Roundup (Monsanto/Bayer, Leverkeusen, Germany) [11,16]. These make it possible to use soil-protective and no-till cultivation methods, significantly reducing pesticide usage. Another successfully used strategy is the cultivation of insect-resistant plants containing the bacterial gene encoding the Bt toxin (Cry protein), that is poisonous for noxious insects [17]. Almost one-third of all genetically-engineered crops have combined herbicide tolerance and insect resistance (stacked genes).

Thus far, crops with genetically-modified genes enhancing resistance to abiotic stresses have not been cultivated on a large scale. This situation results from the fact that most abiotic stress tolerance traits are controlled by multiple genes present at multiple loci [18,19]. However, genome-editing tools, particularly the CRISPR/Cas9 technology, provide the premise for multiple and efficient target modifications of the plant genome [20]. Additionally, faster and cheaper sequencing techniques, such as Next-Generation sequencing of crop genomes, enables the identification of multiple genes involved in responses to abiotic stresses [21]. The coupling of these techniques with traditional and advanced breeding practices could revolutionize plant biotechnology in the coming years.

In this review, we discuss the benefits of modification to genes involved in the regulation of programmed cell death (PCD) and acclimation to biotic and/or abiotic stresses, with a focus on Lesion Simulating Disease 1 (LSD1), Enhanced Disease Susceptibility (EDS1) and Phytoalexin Deficient 4 (PAD4) that have orthologs in many plant species, including *Arabidopsis thaliana, Nicotiana betnhamiana*, *Populus tremula x tremuloides*, *Oryza sativa* [22,23], *Triticum aestivum* [24,25], *Gossypium barbadense* [26,27], *Vitis vinifera* [28,29], *Lycopersicon esculentum* [30,31], *Pisum sativum* [32] and others. Although the potential benefits of *lsd1*, *eds1* and *pad4* mutants over wild type plants have been previously addressed [33,34,35], here we discuss new discoveries related to the applications of *LSD1*, *EDS1* and *PAD4* in improving crops and woody plants for different biotechnological applications.

## 2. LSD1, EDS1, and PAD4 Play a Crucial Role in the Response to Both Biotic and Abiotic Stresses

LSD1 is known as a negative regulator of PCD, since it was found that the *Arabidopsis thaliana lsd1* mutant exhibits an inability to stop cell death once it was activated [36]. Under unfavorable conditions, the phenotype of *lsd1* is manifested by a fast spread of lesions i.e., a runaway cell death (RCD). The RCD phenotype is induced by a superoxide anion (O_2_^−^) and H_2_O_2_, which are excessively produced in an *lsd1* mutant [37,38,39,40,41,42,43,44]. Hormones important for plant defense i.e., salicylic acid (SA) and ethylene (ET) are also over-accumulated in *lsd1* [35,40,42,44,45]. It was found that in the NahG/*lsd1* plants that are unable to accumulate SA because of the NahG bacterial gene, encoding salicylate hydroxylase that converts SA to catechol, cell death was suppressed [46]. Similarly, a mutation in the ET receptor *AtEIN2*, introduced into the *lsd1* background, mitigated the cell death phenotype in *lsd1/ein2* double mutants [47]. These results suggest that LSD1-dependent cell death is associated not only with ROS, but also requires SA and ET function.

LSD1 plays an important role in the response and acclimation of plants to a broad range of stresses such as drought [35,48], UV-C radiation [44,46], cold stress [49], excess light [41], root hypoxia [42] and pathogens [43]. Interestingly, the *lsd1* phenotype shows conditional dependence on environmental conditions, and the morphology and seed yield of the mutant does not differ from the wild type when plants are grown under field conditions [35,45]. LSD1 acts as a negative regulator of EDS1 and PAD4 [43,44,45,50,51] in PCD, and double mutants *eds1/lsd1* and *pad4/lsd1* demonstrate a reverted RCD phenotype, even under stress conditions [44,45].

EDS1 and PAD4 proteins function in resistance (*R*) gene-mediated and basal disease resistance, and can physically interact with each other [52,53]. Both EDS1 and PAD4 show sequence homology to eukaryotic acyl lipases [54,55]. The complexes of EDS1 and PAD4 regulate PAMP-triggered immunity (PTI) as well as Toll–interleukin-1 receptor–nucleotide binding-leucine-rich repeat (TIR-NB-LRR) protein-mediated signaling in response to pathogens [56,57,58]. The EDS1–PAD4 complex is also required for the accumulation of SA and systemic-acquired resistance (SAR) [52]. Mutations in *AtEDS1* and *AtPAD4* result in impaired SA, ET and ROS homeostasis, disrupted acclimatory responses and cell death signaling [38,42,50,51,54]. However, the role of EDS1 in promoting cell death under biotic and abiotic stresses appears superior to PAD4, and a mutation in *AtEDS1* reverses the *lsd1* mutant cell death phenotype stronger than the mutation in *AtPAD4* [43,45]. It was found that LSD1, EDS1 and PAD4 are involved in lysigenous aerenchyma formation [42]. Aerenchyma formation is an acclimation response that allows plants to survive a low availability of oxygen in the soil, for example during flooding [59]. This process is under ROS and ET control, and it was found that in *eds1* and *pad4* mutants with lower ET and ROS content, the percent of the secondary xylem core was smaller than in the *lsd1* mutant with higher ET and ROS content [42]. Numerous studies demonstrate that LSD1, EDS1 and PAD4 form a specific hub that regulates cell death and acclimation responses to both, biotic and abiotic stresses [28,41,42,43,44,46,50,51].

## 3. LSD1, EDS1 and PAD4 Molecular Properties

The LSD1 protein possesses three zinc (Zn)-finger domains, which enable DNA or protein binding [51,60,61]. The Zn-finger motif in LSD1 contains the conserved consensus sequence: CxxCRxxLMYxxGASxVxCxxC that belongs to the C2C2 class [62]. In *Arabidopsis thaliana,* LSD1 was found in both the nucleus and cytoplasm [50]. However, another study on *Pisum sativum* showed only a nuclear localization of LSD1 [32]. So far LSD1 has been found to interact directly or indirectly with 33 proteins [50,63,64,65,66,67,68,69], acting as a scaffold protein. Importantly, these interactions depend upon the cell’s oxidative status [50]. Moreover, it was shown that LSD1 can act as a transcriptional regulator [50]. Significant changes in the transcriptome of the *lsd1* mutant grown under field conditions were found in comparison to the laboratory conditions [35], underlining the conditional-dependent role of LSD1.

Recently it was shown that LSD1 interacts directly with EDS1 [50], while EDS1 forms hetero-dimers with PAD4 [53]. EDS1 is mostly present in the cytoplasm, whereas EDS1–PAD4 and EDS1–LSD1 complexes appear in the nucleus [70]. This suggests dynamic interactions between EDS1 and its signaling partners in the plant cell [50,52,53]. Co-interactions of these proteins appear to be important for their cellular function [53,71]. Moreover, EDS1 interacts with seven other proteins including AT3G48080, considered to be a defense response protein [70], Response to Low Sulfur 1 (LSU1) [72], Recognition of Peronospora Parasitica 8 (RPP8) [73], Resistant to *Pseudomonas syringae* 4 (RPS4) [74], Ribsomal Protein S6 (RPS6) [75], Suppressor OF NPR1-1 (SCN1) [76], and Suppressor OF RPS4-RLD 1 (SRFR1) [77], while PAD4 has been found to interact only with AT3G48080 [71].

## 4. The LSD1, EDS1, and PAD4 Regulatory Hub Links Plastoquinone, Salicylic acid, Ethylene, and ROS Signaling in *Arabidopsis thaliana*

Stress factors, such as high light, UV radiation and high temperature have an influence on the quantum-redox status of the photosynthetic electron transport chain (ETC) components [78], and thus on ROS production and the glutathione/ascorbate redox status. The resulting changes in redox status affect hormone levels (including SA), ion and sugars signal transduction pathways [79,80]. As a feedback, these signals can lead to stomata closure, induce photorespiration, and trigger the overproduction of cell-death-signaling molecules, e.g., ROS and ET [48,79,80,81,82,83,84,85,86]. Importantly, ROS, ET, and SA signaling during cell death are under the control of LSD1, EDS1 and PAD4 [35,51,53,87].

The *lsd1* mutant exhibits lower stomatal conductance, lower catalase (CAT) activity, and higher H_2_O_2_ content, even under control conditions of short-day and low light [35,46]. In response to high light or long-day conditions, *lsd1* over-accumulates H_2_O_2_, ET and SA, that induce RCD [41,42]. 

**Figure 1:**
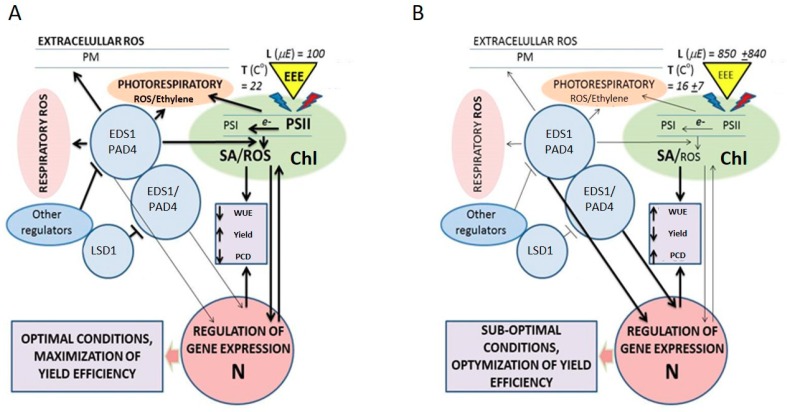
Proposed models of regulation and integration of seed yield, maximal photosynthetic efficiency, reactive oxygen species (ROS)/hormonal cellular homeostasis and water use efficiency by LESION SIMULATING DISEASE 1 (LSD1)/ENHANCED DISEASE SUSCEPTIBILITY 1 (EDS1)/PHYTOALEXIN DEFICIENT 4 (PAD4) in *Arabidopsis*. LSD1/EDS1/PAD4 is proposed to function as a regulatory hub in laboratory (**A**) and field (**B**) conditions. Bold lines—strong regulation, thin lines—weak regulation. WUE—water use efficiency, Yield—seed yield. Average light intensity (µmol of photons m^−2^s^−1^) and temperature (°C) are given on the triangle borders that symbolize the capacity of the photosystems to absorb excess light energy (EEE). Chloroplast (Chl), nucleus (N), photosystem II and I (PSII and PSI), plasma membrane (PM), reactive oxygen species (ROS) and salicylic acid (SA).

It was found that a mutation in the *Chrloroplast Signal Recognition Particle 43 (AtCAO)* gene rescues the *lsd1* phenotype. The *cao/lsd1* double mutant absorbs fewer photons due to smaller photosystem II antennas size and higher NPQ, and this leads to an inhibition of RCD in response to excess light, compared to the *lsd1* single mutant [41]. Therefore, the RCD phenotype of *lsd1* is linked to the amount of absorbed light and redox status of plastoquinone (PQ) [51]. *lsd1* also exhibits an RCD phenotype in response to UV stress [44], and it is known that UV irradiation affects the photosynthetic apparatus and redox status of the PQ pool [88,89]. LSD1 conformation and its activity are controlled by changes in the PQ pool [64], and it was hypothesized that under stress conditions, changes in the activity of LSD1 can reduce the activity of SOD and CAT proteins, and downregulate *PR1* gene expression [51] (Figure 1). Under non-stress laboratory condition, *eds1* and *pad4* mutants accumulate less H_2_O_2_ than wild type plants [45], while double *eds1/lsd1* and *pad4/lsd1* exhibit similar [35] or lower [41] H_2_O_2_ level than wild type.

*Arabidopsis thaliana* EDS1 and PAD4 form a regulatory hub, influencing the accumulation of SA [45,90]. Moreover, SA induces stomatal closure [91], which leads to photoinhibition [92,93,94]. Both inhibition of the antioxidant system and photoinhibition cause higher ROS accumulation [78,95,96,97,98]. It was found that *eds1* and *pad4* mutants accumulate less SA than the wild type during biotic stress, while there is no difference in SA accumulation in plants overexpressing EDS1 or PAD4 [90]. Importantly, *Arabidopsis* plants overexpressing both EDS1 and PAD4 accumulate significantly more SA than wild type in response to biotic stress [87].

## 5. Involvement of Salicylic Acid, Ethylene, and ROS in Plant Productivity 

LSD1, EDS1 and PAD4 can regulate SA, ET and ROS metabolism during systemic acquired acclimation [41,42] and systemic acquired resistance [43].

High foliar SA and H_2_O_2_ levels were found in the *lsd1* mutant under both control and stress conditions. EDS1 and PAD4 mediate the ROS-derived signal leading to RCD in the *lsd1* mutant [35,42]. LSD1 is also required for the SA-dependent induction of the antioxidant enzyme copper-zinc superoxide dismutase (Cu–Zn SOD) and potentially other antioxidant genes [33,41,44]. 

A ROS level exceeding the capability of the plant cell to mount an effective antioxidant response is harmful and could damage DNA, RNA, proteins and membranes, and in extreme cases, cause plant death [81,98]. However, H_2_O_2_ and O_2_^−^ and their derivatives are also natural by-products of cellular metabolism, and are involved in a broad range of plant physiological and biochemical processes i.e., signaling, defense response and photosynthesis regulation during the entire plant lifespan [98,99,100]. Reactive Oxygen Species (ROS) do lead to growth inhibition and lower plant productivity during biotic and abiotic stress. Therefore, deregulation of genes involved in ROS metabolism or signaling (inclulding *LSD1*, *EDS1* and *PAD4*) also affects plant growth and development [44,45,101,102,103].

Similarly, high levels of SA that accumulate during plant responses to environmental stresses are close to phytotoxicity, and could reduce plant growth or induce cell death [104,105,106]. However, through a complex signaling network, SA plays an important role in seed germination, root initiation, floral induction and thermogenesis [107,108,109]. SA is a negative regulator of auxins, and interferes with auxin-mediated responses [108,109]. In support of this link, SA-accumulating dwarf mutants such as *cpr5*, *cpr6*, and *snc1* were shown to contain lower endogenous levels of free IAA and reduced sensitivity to auxins, compared with wild-type plants [110]. Moreover, the effects of the *cpr1* and *cpr6* mutations on SA-related gene expression were dependent upon PAD4 function, while SA accumulation in the lesion-mimic mutant *cpr5* was partially PAD4-independent, and in other dwarf mutants, such as *dnd1* and *dnd2*, it was completely PAD4-independent [111]. The significance of SA in plant growth is also demonstrated by the fact that the expression of bacterial NahG, which decomposes SA to katehol, in *lsd1* or *acd6* (Accelerated Cell Death 6) backgrounds reverts their dwarf phenotypes [46,112]. Recent studies on *Arabidopsis thaliana* have also shown that SA content in plant tissues is strongly correlated with seed yield [45]. Accordingly, mutations in genes encoding positive regulators of SA signaling, EDS1 and PAD4, revert impaired growth and seed yield in the *lsd1* mutant [35,45]. Further, the EDS1-PAD4 complex is required for SA accumulation [52] and SA causes the up-regulation of EDS1 and PAD4 expression [113]. However, the SA role in plant productivity is not clear, since exogenous treatment with a low concentrations of SA increases the growth of soybean [114], wheat [115,116] and maize [117], while exogenous SA treatment with a higher SA concentration can inversely influence plant growth [118]. The impact of SA levels on plant growth and biomass production is presented in Table 1.

It has been shown that the ROS levels in plant tissues are highly interconnected with ET [82,104], and cooperate in the regulation of plant productivity [122]. ET as a gaseous hormone can freely enter plant cells [123]. It regulates many aspects of plant physiological processes, such as ripening, abscission, vegetative development, senescence and response to stress [123,124,125]. In both shoots and roots, ET causes the inhibition of cell expansion, affecting plant growth [126,127] and biomass production [128]. EDS1 and PAD4 operate upstream of ET and ROS production during light stress responses, and together with LSD1 regulate the signaling of PCD, light acclimation and defense responses through redox changes of the PQ pool [47]. Mutant analysis confirms that LSD1 suppresses EDS1- and PAD4-dependent ROS and ET signaling, and that EIN2, the ET signal transduction protein, acts downstream of EDS1 and PAD4 [47]. Propagation of PCD in the *lsd1* mutant is also dependent upon EIN2, since the *ein2/lsd1* double mutant has significantly reduced runaway cell death, compared with *lsd1* under stress conditions [47].

Further evidence points to an important role for photosynthesis, chloroplasts and the redox status of the PQ pool in regulating LSD1-, EDS1- and PAD4-modulated stress responses [51]. Plants evolved unique mechanisms that depend upon excess excitation energy and redox (including ROS) signaling, originating at the chloroplast. Such response systems are likely to be crucial for plant growth, biomass production and their development in the natural environment. Naturally occurring changes in light intensity, temperature and humidity make plant growth dependent on successful acclimation to fluctuating conditions. Taking into consideration the research described above, the LSD1/EDS1/PAD4 hub may be a key regulator of plant acclimation and defense responses, as well as potentially, plant growth and development.

## 6. LSD1, EDS1, and PAD4 are Involved in Biomass Production, Seed Yield Regulation, and Water Use Efficiency in *Arabidopsis thaliana*

Acclimation and defense responses are regulated by redox sensing and modifications in the proximity of photosystem II and non-photochemical quenching (NPQ), the redox status of glutathione and plastoquinone pools, photoelectrophysiological signaling, ROS metabolism, hormonal circuits and cellular light stress memory [52,79,98,129,130,131,132]. Disturbances or changes in these processes may significantly affect the balance between plant cell death and cell division processes, and thus acclimation responses, biomass production and growth, these being processes that are under the control of LSD1, EDS1 and PAD4 [48,50,51].

From the moment of its discovery, the *lsd1* mutant was described as a dwarf [36], due to *lsd1* rosette size, that in non-permissive conditions is significantly smaller compared to wild-type plants. Moreover, dry biomass of *lsd1* was significantly lower than wild type [48]. Interestingly, no significant differences were found between the biomass of *lsd1* mutants and wild-type plants under field conditions [45]. 

Under non-permissive conditions (long photoperiod, UV-C episode) the *lsd1* mutant produced less biomass than wild-type, *eds1,* or *pad4* mutants [33,45,87]. Mutations in *AtEDS1* and *AtPAD4* result in a reversal of the *lsd1* phenotype, since double *eds1/lsd1* and *pad4/lsd1* mutants do not differ from the wild type in terms of biomass production [45].

The LSD1/EDS1/PAD4 hub is not only involved in vegetative growth regulation, but also in the regulation of inflorescence development and seed production. Under laboratory conditions, reduced inflorescence growth and lower seed yield were found in *lsd1,* but not in double *eds1/lsd1* and *lsd1*/*pad4* mutants [35,45]. Surprisingly, this phenomenon was not observed under field conditions, in which the inflorescence and seed yield were similar between wild type, *lsd1*, *eds1*, *pad4*, *eds1/lsd1* and *pad1/lsd1* mutants [35,45]. It was proposed that under natural conditions, the LSD1 suppressive role of cell death and its role in growth regulation are dependent upon other, yet unknown regulators. The fact that *lsd1* mutants differ from the wild type in terms of biomass, inflorescence development and seed yield in the laboratory, but not in the field [35,45], indicates that LSD1, EDS1 and PAD4 conditionally regulate the signaling pathways engaged in plant development.

The amount of biomass produced by plants is determined, among others, by the availability of water [133]. Although the role of LSD1, EDS1 and PAD4 in the regulation of water use efficiency (WUE) is not clear, it was found that the *lsd1* mutant produces significantly less dry mass per 1 mL of water utilized, in comparison to the wild type under field conditions, whereas double mutants *eds1/lsd1* and *pad4/lsd1* exhibit similar WUE values to the wild type. Under laboratory conditions, no differences were found between *lsd1* and the wild type [35]. On the other hand, water loss, corresponding to transpiration, was reduced in both laboratory- and field-grown *lsd1* mutants, compared to wild type [48]. Lower water requirements dependent upon LSD1 could be only partly explained by lower stomata density, because a noticeable decrease in stomata number seems to have an effect only on *lsd1* plants grown under stable laboratory conditions, but not under multivariable field conditions [48]. Therefore, other mechanisms should be considered in explaining this LSD1-dependent control of water loss (stomatal regulation), and its utilization. SA, ET and H_2_O_2_, which are important during photosynthesis and the control of stomatal conductance [134,135,136,137], can be potential candidates. Indeed, mathematical models confirm that LSD1, EDS1 and PAD4, together with SA and H_2_O_2_, are involved in the regulation of water use efficiency (WUE) and vegetative and generative development [45]. Additionally, a strong correlation between SA and H_2_O_2_ content in 4-week-old plants and seed yield, determined for 9-week-old plants, may indicate that mechanisms dependent upon LSD1, EDS1 and PAD4 are involved in the algorithmic computation performed by plants, in order to optimize ROS and hormone content at early stages of plant development, in order to ensure maximal seed yield.

In summary, LSD1, EDS1 and PAD4 constitute a signaling hub, which integrates plant responses to water stress, vegetative biomass production and generative development. This hub plays a significant role in at least in two different species, *Arabidopsis thaliana* and *Populus tremula × P. tremuloides.*

## 7. LSD1, EDS1, and PAD4 Regulate Morphology, Photosynthetic Efficiency, and Wood Properties in *Populus tremula* L. × *P. tremuloides*

Poplar, referred to as the “*Arabidopsis* of forestry” [138], is widely used for the study of molecular mechanisms, as well as for industrial applications, mainly paper and bioethanol production [139,140]. Thanks to the availability of genetic engineering methods, new poplar genotypes with improved growth and biomass accumulation were created [38,107,121,141,142]. Biomass accumulation in woody plants is strictly related to cell division at the primary and secondary meristems [143]. Therefore, plant growth and biomass production can be improved by an overall increase in cell number [144,145,146], which is under the control of genes involved in cell cycle regulation [147,148] or hormone biosynthesis [149,150].

**Figure 2:**
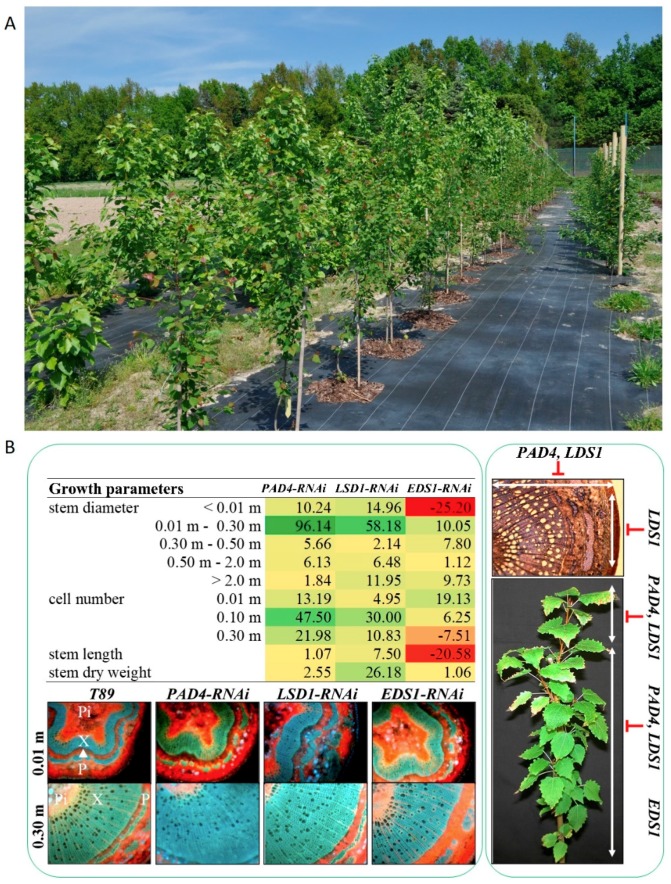
Photograph of transgenic poplar plantation (**A**), and the regulation of tree growth by LSD1, EDS1 and PAD4 (**B**). Percentage changes in growth parameters are presented in relation to wild-type trees (T89). Lower expression of *LSD1* and *PAD4*, but not *EDS1*, increased biomass accumulation, measured as stem length and diameter, stem dry weight and xylem accumulation. Higher cell numbers and improved diameters were observed in young parts of the stems of *PAD4*-*RNAi* trees, while *LSD1*-*RNAi* trees also displayed a higher diameter of the older part of the stem, accompanied by higher stem length. Fluorescent microscopy of stem cross-sections demonstrate: *Pi* = pith, *X* = xylem; *Δ* = cambium, *P* = phloem. Based on these results the following model was suggested: PAD4 deregulates (inhibits) periclinal cell division. LSD1 has a significant effect upon both periclinal and anticlinal divisions, on cell elongation and differentiation, while EDS1 is a positive regulator of cell division and differentiation in poplars [38,48].

Down-regulation of *LSD1, EDS1,* and *PAD4* affects poplar growth and biomass accumulation [48]. In transgenic poplar lines *LSD1-RNAi, EDS1-RNAi* and *PAD4-RNAi* were found a higher number of cells, but only in *PAD4-RNAi* and *LSD1-RNAi* lines did it result in larger stem diameter and stem length [48]. Nevertheless, a greater number of lateral shoots was observed in *EDS1-RNAi* lines, and this may also result in improved total biomass production [38]. Furthermore, *LSD1, EDS1* and *PAD4* influence wood structure. The cell wall was thicker in all *LSD1*-, *EDS1*- and *PAD4*-silenced lines, among others as a result of changes in the cell wall composition. The increase in hemicelluloses, decline in cellulose, and reduced lignin quantity and stability in the *LSD1-RNAi* wood, resulted in easy wood degradation, which may be important for its improved industrial application. In contrast, the cell wall of *PAD4-RNAi* trees had more cellulose and lignin, and less hemicelluloses, and was more resistant to degradation (Figure 2).

These changes may be linked to higher H_2_O_2_ content in poplar transgenic lines [38,121], since ROS are important in the development of the cell wall [86,151,152,153,154]. H_2_O_2_ is involved in the differentiation of secondary cell walls and in the polymerization of cinnamyl alcohols in lignifying xylem vessels [155,156]. Moreover, SA is an important component of signaling pathways engaged in plant cell growth [157]. Lower levels of SA were found in poplar lines with silenced *EDS1* [38]. These results suggest that it is possible to engineer the plant cell-walls without negative impact on their growth and development. From the wood industry point of view, i.e., the production of paper and bioethanol, material obtained from such wood is cheaper, and its production is less harmful for the environment [158].

Since biomass production is tightly dependent upon photosynthesis [159], it is not surprising that producing more abundant lateral shoots in the *EDS1-RNAi* lines correlates with improved photosynthetic parameters (quantum yield of photosystem II, photochemical and non-photochemical quenching), chlorophyll content, and thus photosynthetic efficiency. Moreover, *EDS1-*silenced trees have a higher CO_2_ assimilation and photosynthetic capacity and maintain their leaves longer, since processes involved in senescence are delayed [38].

## 8. Orthologs of LSD1, EDS1, and PAD4 in Crops

Orthologs of *LSD1, EDS1* and *PAD4* were found in many plant species, including crops and industrial plants (Table 2). While most model plants are dicotyledonous, many crop plants are monocotyledonous. However, it was postulated that despite some clade/species-specific changes, an evolutionarily-conserved regulon containing core components of plant innate immunity is present [160]. LSD1, EDS1 and PAD4 originated prior to the differentiation of monocots and dicots [24]. Despite some structural differences, LSD1, EDS1 and PAD4 seem to have a similar function in all plant species where they are identified. The different crops described below are of great importance in agriculture, and thus genes involved in their resistance, biomass production, productivity and development are important from the agricultural point of view.

Rice (*Oryza sativa*) is one of the most important crops, and is a crucial dietary component for over 3.5 billion people [161]. Approximately 480 million metric tons of rice is produced annually [162]. Rice was the first fully-sequenced crop [163], which makes genetic studies of rice relatively easy. Orthologs of *AtLSD1, AtEDS1* and *AtPAD4* were found in rice [22,23] (Table 2). Similarly to *Arabidopsis*, *LSD1* in rice is light-induced and dark-suppressed [23,41,47]. In *LSD1* antisense transgenic rice, a higher expression of *PR1* and a lesion phenotype was observed, which indicates that LSD1 is a negative regulator of cell death in rice [23]. Expression of *OsLSD1* in *Nicotiana tabacum* was shown to enhance resistance to mycotoxins [23]. Furthermore, the regulation of PCD by OsLSD1 occurs through direct interactions of LSD1 with rice metacaspase (OsMC) [164], similar to *Arabidopsis*, where *At*LSD1-dependent cell death is associated with metacaspase activity [60]. To date, no studies were performed on mutants or transgenic crop plants with deregulated expression of the *OsEDS1*. However, the expression of *OsEDS1* was shown to be upregulated in response to biotic stress and SA treatment [165,166]. Interestingly, the function of OsPAD4 is different from AtPAD4 [22]. While AtPAD4 is localized in the cytoplasm and nucleus [50,167], *OsPAD4* encodes a plasma membrane protein [22]. Furthermore, the AtPAD4-induced pathway against biotrophic pathogens is SA-dependent, while OsPAD4 is involved in JA-dependent defense responses [22]. *OsPAD4*-silenced plants exhibit enhanced susceptibility to biotrophic pathogens associated with impaired accumulation of jasmonic acid (JA) and phytoalexinmomilactone A (MOA) [22]. Moreover, exogenous JA application complemented the susceptibility phenotype of *OsPAD4*-silenced rice [22].

Grape (*Vitis vinifera*) is an important fruit plant with world production of about 27 million tons [168]. This plant is susceptible to infection, especially by fungi [28,169,170,171,172,173]. However, there is currently no information about *LSD1* orthologs in *Vitis vinifera*, or also studies were not carried out on grapes with deregulated *VvLSD1* expression, or even the *VvLSD1* expression profile was not investigated. In contrast, *Vitis vinifera* orthologs of *AtEDS1* and *AtPAD4* have been studied in grape [28,29,174,175], and *EDS1-LIKE* (*EDL*) genes were found in the grape genome [29]. There is a molecular evidence that EDS1 and PAD4 form a stable complex in grape, which supports the SA defense pathway in response to biotic stress [29,175]. However, it was postulated that the EDS1/PAD4 hub in grape is more complicated, compared to *Arabidopsis* [29]. The expression of *VvEDS1* and *VvEDL* was up-regulated by pathogens [28,174] and that of *VvEDS1* additionally by SA [29]. *VvEDS1* expression complemented the *Arabidopsis eds1* phenotype, whereas the expression of *VvPAD4* did not complement the *pad4* mutant phenotype. Interestingly, VvEDS1 and VvEDL proteins can interact not only with VvPAD4, but also with AtPAD4, while VvPAD4 cannot interact with AtEDS1 [29]. These results support a similar role for EDS1 in grape and *Arabidopsis*, but also show some differences between AtPAD4 and VvPAD4.

Cotton (*Gossypium barbadense*) is widely used in textile, feed, food, biooil and biofuel production [176]. Cotton crops are susceptible to pathogens such as *Verticillium dahlia,* which significantly reduces yield [177,178]. An ortholog of AtEDS1 was found in cotton with an amino acid sequence similarity of 46%, 53%, and 54% with *Arabidopsis thaliana, Solanum lycopersicum* and *Nicotiana benthamiana*, respectively. Similarly to EDS1 orthologs from other species, GbEDS1 contains a conserved N-terminal lipase domain and an EDS1-specific KNEDT motif, and is localized to both the cytoplasm and nucleus [26]. *GbEDS1* expression is drastically increased in response to pathogens [26,27]. Moreover, overexpression of *GbEDS1* triggers higher SA and H_2_O_2_ production, while its silencing results in significantly-decreased SA and H_2_O_2_ accumulation [27]. It was found that orthologs of *AtPAD4* were up-regulated in cotton during pathogen infection [178]. So far, no data describing the role of LSD1 in cotton was shown.

A functional homolog to *AtLSD1* and orthologs of *AtEDS1* were found in *Triticum aestivum*. [24,25]. *TaLSD1* encodes 146 amino acid long protein, which contains three zinc-finger domains, similarly to AtLSD1 [61]. Generally, TaLSD1 is a regulator of cell death and is involved in disease resistance of wheat against pathogens [25]. This suggests that the role of *At*LSD1 and *Ta*LSD1 is similar [43,61]. The expression of *TaLSD1* is up-regulated during interaction with the fungal pathogen, *Puccinia striiformis,* and in response to oxidative stress-generating compound methyl viologen [25]. Using an onion epidermal system, TaLSD1 was found in the nucleus [25]. Interestingly, *TaLSD1* overexpression in *Nicotiana benthamiana* inhibits cell death induced by the expression of the mammalian *BAX* gene, which encodes a regulator of apoptosis [179,180,181]. Moreover, silencing of *TaLSD1* in wheat resulted in higher *TaPR1* expression and enhanced hypersensitive response [25]. *TaEDS1* was also described recently in wheat [24]. Three orthologs of *AtEDS1* were found in the *Triticum aestivum* genome (*TaEDS1*-*5A*, *5B* and *5D*). The similarity between these *TaEDS1* cDNA sequences was greater than 96%. Moreover, *TaEDS1*-*5A*, *5B* and *5D* genes share sequence similarity of about 99% with the respective diploid ancestor orthologs. *TaEDS1* is strongly up-regulated in response to pathogen and SA treatment [24]. Most importantly, the expression of *TaEDS1* in the *eds1* mutant complemented its susceptible phenotype to powdery mildew [24].

Orthologs of *AtLSD1* or *AtEDS1* have been also found in crops such as *Solanum melongena, Lycopersicon esculentum, Pisum sativum, Glycine max* and *Solanum tuberosum* [30,32,182,183,184]. LeEDS1 is involved in SA accumulation since tomato *eds1* mutants have significantly reduced SA level and impaired R gene-mediated resistance to viral, bacterial, and fungal pathogens [30]. It was also reported that tomato EDS1 is involved in both basal and *R* gene-mediated resistance [30,31], and its expression is up-regulated in response to pathogen infection [185,186]. Moreover, it was found that *LePAD4* expression is up-regulated in response to green peach aphid [187]. A single copy of *SmEDS1* was mined from the *Solanum melongena* genome draft. Using bioinformatic methods, it was determined that the full-length *SmEDS1* gene is 4.5kb long and contains three exons coding for 1.8kb mRNA. The described gene encodes a protein consisting of 602 amino acids [184]. 

The LSD1 protein has been well characterized in *Pisum sativum* [32]. It was found that PsLSD1 is involved in PCD regulation and the overexpression of *PsLSD1* in the *Arabidopsis lsd1* mutant reverts the RCD phenotype in response to SA treatment [32]. It was shown that PsLSD1 is a nuclear-localized protein containing zinc finger motifs, and can act as a transcription regulator [32], similar to AtLSD1 [50]. The *Glycine max* genome encodes two EDS1 isoforms and one PAD4 ortholog. GmEDS1 and GmPAD4 are necessary for defense signaling in *Glycine max*, and their structure is similar to AtEDS1 and AtPAD4, respectively. Moreover, the expression of *GmEDS1* and *GmPAD4* in *eds1* and *pad4* mutants complemented their pathogen-resistant phenotypes [188], while the expression of *GmEDS1* and *GmPAD4* in *eds1/pad4* double mutant did not complement pathogen-induced SA accumulation [188]. Importantly, transgenic soybean lines with silenced *GmEDS1* or *GmPAD4* demonstrated reduced basal and pathogen-induced SA accumulation and increased susceptibility to virulent pathogens [188]. GmLSD1 is involved in response to biotic stress, and GmLSD1 is a negative regulator of cell death [183]. In addition, genomic sequences of *StEDS1* and *StPAD4* were obtained from the potato genome [182]. All those studies seem to be a great starting point for future research, cloning and expression analysis of *LSD1, EDS1* and *PAD4* genes.

## 9. Future Perspectives

The role of LSD1, EDS1 and PAD4 is well known in model plants, such as *Arabidopsis thaliana*, *Nicotiana benthamiana* and *Populus tremula × P. tremuloides*, but some mechanism and dependencies are still unknown. The function of LSD1, EDS1 and PAD4 appears to be highly conserved, and is similar in many plant species, both monocots and dicots. Because of their high importance, the function of these proteins should be studied further in model plants, and new knowledge should be transferred to crops and industrial plants.

In this article, we described different examples suggesting that manipulating *LSD1, EDS1* and *PAD4* could be beneficial and useful for enhanced agriculture production. To date, no studies have been performed on crops with modified expressions of *LSD1*, *EDS1* and *PAD4* orthologs in the context of yield and biomass production. Interestingly, the role of *AtPAD4* orthologs in crops seems to be different from its role in model plants [22]. Precise determination of the role of LSD1, EDS1 and PAD4 in the regulation of plant productivity and their role in regulating SA/ROS homeostasis may allow faster crop selection in the future. Climate change impacts also involve a reduction in the availability of water resources. Therefore, the role of proteins, that can participate in the regulation of the WUE, such as LSD1, EDS1 and PAD4, should be studied. 

Climate change causes not only stronger and more frequent abiotic stress, such as heat, high light or drought, but also more intense biotic stresses. It is estimated that in the near future, losses to agricultural production associated with excessive stress may increase by 10–25%. LSD1, EDS1 and PAD4 have been described as universal regulators of responses to both biotic and abiotic stresses in *Arabidopsis thaliana,* but also potentially in crops. Therefore, manipulating their expression could be an interesting alternative for adapting different crop varieties to climate change. The different LSD1, EDS1 and PAD4 mutants have also been used as an example in the development of algorithms to determine future yield at early stages of cultivation, based upon some physiological traits. Implementing such algorithms could significantly accelerate the breeding process and reduce the cost of agricultural production. Manipulating genes involved in responses to biotic stress in order to improve plant yield or biomass production may carry the risk of greater susceptibility to pathogens. However, our 10 year-long field experiment with poplar and *Arabidopsis thaliana* did not show increased susceptibility to pathogens in any of the mutants or silenced lines tested.

## Figures and Tables

**Table 1:** Effect of mutation, gene silencing or bacterial genes expression in plant on SA level and plant phenotype.

Organism	Mutation, Transgene or Gene Silencing	Effect on SA Level	Growth Phenotype	Reference
*Arabidopsis thaliana*	Bacterial NahG expression	Lower level of SA in transgenic plants	Higher biomass, higher seed yield	[106]
*Arabidopsis thaliana*	Mutation in *ICS1*	Lower level of SA in the mutant	Higher biomass, higher seed yield	[106]
*Arabidopsis thaliana*	Mutation in *CPR1*	A significantly higher level of SA in the mutant	Dwarf phenotype	[119]
*Arabidopsis thaliana*	Mutation in *LSD1*	A significantly higher level of SA in the mutant	Lower seed yield	[35]
*Arabidopsis thaliana*	Mutation in *MPK4*	A significantly higher level of SA in the mutant	Dwarf phenotype	[120]
*Populus tremula x tremuloides*	Lower expression of *PAD4*	Lower level of SA in transgenic lines	Higher stem diameter, higher % of dry weight	[48,121]
*Populus tremula x tremuloides*	Lower expression of *EDS1*	Lower level of SA in transgenic lines	Higher CO_2_ assimilation, changed plant morphology	[38]
*Populus tremula x tremuloides*	Lower expression of *MPK4*	Two times higher level of SA in transgenic lines	Lower perimeter of main stem	[107]

**Table 2:** Characterized orthologs of *LSD1*, *EDS1* and *PAD4* in crops.

Ortholog of:	Species	Reference
*AtLSD1*	*Oryza sativa* *Triticum aestivum* *Pisum sativum*	[23] [25] [32]
*AtEDS1*	*Oryza sativa,* *Gossypium barbadense* *Vitis vinifera* *Lycopersicon esculentum* *Triticum aestivum*	[166] [26,27] [28,176] [30,186,188] [24]
*AtPAD4*	*Oryza sativa* *Vitis vinifera*, *Gossypium barbadense*	[22] [176] [179]
